# Supplementary material for: Rehabilitation with and Without Robot and Allied Digital Technologies (RADTs) in Stroke Patients: A Study Protocol for a Multicentre Randomised Controlled Trial on the Effectiveness, Acceptability, Usability, and Economic-Organisational Sustainability of RADTs from Subacute to Chronic Phase (STROKEFIT4)
Source: J Clin Med. 2025 Apr 15;14(8):2692. doi: 10.3390/jcm14082692 (PMC12028101; doi:10.3390/jcm14082692)
Supplement: Supplementary file 1 [file jcm-14-02692-s001.zip › jcm-3523947-supplementary.pdf]

Satisfaction with Rehabilitation Services Questionnaire

(At baseline, to be filled only if the patient has undergone at least one week of rehabilitation treatment)

1. ***How satisfied are you overall with the current provision of rehabilitation services?***
   - ☐ 1 (not satisfied)
   - ☐ 2
   - ☐ 3
   - ☐ 4
   - ☐ 5 (very satisfied)
2. ***How satisfied are you with the material infrastructures (facilities, buildings, dedicated areas, etc.)?***
   - ☐ 1 (not satisfied)
   - ☐ 2
   - ☐ 3
   - ☐ 4
   - ☐ 5 (very satisfied)
3. ***How satisfied are you with the immaterial infrastructures (connection, telemedicine or tele-rehabilitation, etc.)?***
   - ☐ 1 (not satisfied)
   - ☐ 2
   - ☐ 3
   - ☐ 4
   - ☐ 5 (very satisfied)
4. ***How satisfied are you with the communication between healthcare provider and patient/family?***
   - ☐ 1 (not satisfied)
   - ☐ 2
   - ☐ 3
   - ☐ 4
   - ☐ 5 (very satisfied)
5. ***How urgently should the material infrastructures (facilities, buildings, dedicated areas, etc.) be improved?***
   - ☐ 1 (not urgent)
   - ☐ 2
   - ☐ 3
   - ☐ 4
   - ☐ 5 (immediately)
6. ***How urgently should the immaterial infrastructures (connection, telemedicine or tele-rehabilitation, etc.) be improved?***
   - ☐ 1 (not urgent)
   - ☐ 2
   - ☐ 3
   - ☐ 4
   - ☐ 5 (immediately)
7. ***How urgently should the communication between healthcare provider and patient/family be improved?***
   - ☐ 1 (not urgent)
   - ☐ 2
   - ☐ 3
   - ☐ 4
   - ☐ 5 (immediately)

Satisfaction with Rehabilitation Services Questionnaire
(After the intervention – both groups)

1. ***How satisfied are you overall with the current provision of rehabilitation services?***
   - ☐ 1 (not satisfied)
   - ☐ 2
   - ☐ 3
   - ☐ 4
   - ☐ 5 (very satisfied)
2. ***How satisfied are you with the material infrastructures (facilities, buildings, dedicated areas, etc.)?***
   - ☐ 1 (not satisfied)
   - ☐ 2
   - ☐ 3
   - ☐ 4
   - ☐ 5 (very satisfied)
3. ***How satisfied are you with the immaterial infrastructures (connection, telemedicine or tele-rehabilitation, etc.)?***
   - ☐ 1 (not satisfied)
   - ☐ 2
   - ☐ 3
   - ☐ 4
   - ☐ 5 (very satisfied)
4. ***How satisfied are you with the communication between healthcare provider and patient/family?***
   - ☐ 1 (not satisfied)
   - ☐ 2
   - ☐ 3
   - ☐ 4
   - ☐ 5 (very satisfied)
5. ***How urgently should the material infrastructures (facilities, buildings, dedicated areas, etc.) be improved?***
   - ☐ 1 (not urgent)
   - ☐ 2
   - ☐ 3
   - ☐ 4
   - ☐ 5 (immediately)
6. ***How urgently should the immaterial infrastructures (connection, telemedicine or tele-rehabilitation, etc.) be improved?***
   - ☐ 1 (not urgent)
   - ☐ 2
   - ☐ 3
   - ☐ 4
   - ☐ 5 (immediately)
7. ***How urgently should the communication between healthcare provider and patient/family be improved?***
   - ☐ 1 (not urgent)
   - ☐ 2
   - ☐ 3
   - ☐ 4
   - ☐ 5 (immediately)

Satisfaction With Robotic Services Questionnaire

(After the intervention - patients enrolled in the robotic group only)

1. ***How satisfied are you overall with the provision of robotic services?***
   - ☐ 1 (not satisfied)
   - ☐ 2
   - ☐ 3
   - ☐ 4
   - ☐ 5 (very satisfied)
2. ***How satisfied are you with the material infrastructures (facilities, buildings, dedicated areas, etc.)?***
   - ☐ 1 (not satisfied)
   - ☐ 2
   - ☐ 3
   - ☐ 4
   - ☐ 5 (very satisfied)
3. ***How satisfied are you with the immaterial infrastructures (connection, telemedicine or tele-rehabilitation, etc.)?***
   - ☐ 1 (not satisfied)
   - ☐ 2
   - ☐ 3
   - ☐ 4
   - ☐ 5 (very satisfied)
4. ***How satisfied are you with the communication between healthcare provider and patient/family?***
   - ☐ 1 (not satisfied)
   - ☐ 2
   - ☐ 3
   - ☐ 4
   - ☐ 5 (very satisfied)
5. ***How urgently should the material infrastructures (facilities, buildings, dedicated areas, etc.) be improved?***
   - ☐ 1 (not urgent)
   - ☐ 2
   - ☐ 3
   - ☐ 4
   - ☐ 5 (immediately)
6. ***How urgently should the immaterial infrastructures (connection, telemedicine or tele-rehabilitation, etc.) be improved?***
   - ☐ 1 (not urgent)
   - ☐ 2
   - ☐ 3
   - ☐ 4
   - ☐ 5 (immediately)
7. ***How urgently should the communication between healthcare provider and patient/family be improved?***
   - ☐ 1 (not urgent)
   - ☐ 2
   - ☐ 3
   - ☐ 4
   - ☐ 5 (immediately)

TAM Questionnaire

Assign a score from 1 (strongly disagree) to 7 (strongly agree) to the following statements.
NB: The choice between robotic solutions and rehabilitation treatments depends on the randomization group.

Perceived Usefulness

- Using (*robotic solutions/rehabilitation treatments*) tested for rehabilitation would make my rehabilitation easier.

1	2	3	4	5	6	7

- Using (*robotic solutions/rehabilitation treatments*) tested for rehabilitation would help me perform tasks more quickly.

1	2	3	4	5	6	7

- Using (*robotic solutions/rehabilitation treatments*) tested for rehabilitation would increase the quality of my rehabilitation.

1	2	3	4	5	6	7

- Using (*robotic solutions/rehabilitation treatments*) tested for rehabilitation would give me more control over rehabilitation activities.

1	2	3	4	5	6	7

- Using (*robotic solutions/rehabilitation treatments*) tested for rehabilitation would make the execution of tasks required in my rehabilitation more effective.

1	2	3	4	5	6	7

Ease of Use

- My interaction with (*robotic solutions/rehabilitation treatments*) tested for rehabilitation will be clear and understandable.

1	2	3	4	5	6	7

- I find it will be easy to get (*robotic solutions/rehabilitation treatments*) tested for rehabilitation to do what I want them to do.

1	2	3	4	5	6	7

- Overall, I find that (*robotic solutions/rehabilitation treatments*) tested for rehabilitation will be easy to use.

1	2	3	4	5	6	7

- Learning to use (*robotic solutions/rehabilitation treatments*) tested for rehabilitation will be easy for me.

1	2	3	4	5	6	7

Compatibility

- I find that using (*robotic solutions/rehabilitation treatments*) tested for rehabilitation will be compatible with every aspect of my rehabilitation.

1	2	3	4	5	6	7

- I find that using (*robotic solutions/rehabilitation treatments*) tested for rehabilitation will fit well with how I like to perform exercises.

1	2	3	4	5	6	7

- I find that using (*robotic solutions/rehabilitation treatments*) tested for rehabilitation will fit well with the style of my rehabilitation.

1	2	3	4	5	6	7

Intention to Use

- I intend to increase the use of (*robotic solutions/rehabilitation treatments*) tested for rehabilitation in the centers where I am assisted.

1	2	3	4	5	6	7

- I intend to increase the use of (*robotic solutions/rehabilitation treatments*) tested for rehabilitation at home.

1	2	3	4	5	6	7

Complexity of solution

(timing of administration: before rehabilitation)

Rehabilitation

1. ***What is the main problem you encounter during your rehabilitation?***
   - ☐ Self-care
   - ☐ Language
   - ☐ Balance
   - ☐ Walking
   - ☐ Manual skills
   - ☐ Other (specify): ____
2. ***Based on your experience, how complex is this problem?***
   - ☐ 1 (not complex)
   - ☐ 2
   - ☐ 3
   - ☐ 4
   - ☐ 5 (extremely complex)

Conventional rehabilitation solution

3. ***What is the main problem addressed by current conventional rehabilitation solutions?***
   - ☐ Self-care
   - ☐ Language
   - ☐ Balance
   - ☐ Walking
   - ☐ Manual skills
   - ☐ Other (specify): ____
4. ***Based on your point of view, how complex is this problem?***
   - ☐ 1 (not complex)
   - ☐ 2
   - ☐ 3
   - ☐ 4
   - ☐ 5 (extremely complex)
5. ***How complex are current conventional rehabilitation solutions?***
   - ☐ 1 (not complex)
   - ☐ 2
   - ☐ 3
   - ☐ 4
   - ☐ 5 (extremely complex)

Robotic rehabilitation solution

6. ***What is the main problem addressed by robotic rehabilitation solutions?***
   - ☐ Language
   - ☐ Balance
   - ☐ Walking
   - ☐ Manual skills
   - ☐ Other (specify): ____
7. ***Based on your point of view, how complex is this problem?***
   - ☐ 1 (not complex)
   - ☐ 2
   - ☐ 3
   - ☐ 4
   - ☐ 5 (extremely complex)
8. ***How complex are robotic rehabilitation solutions?***
   - ☐ 1 (not complex)
   - ☐ 2
   - ☐ 3
   - ☐ 4
   - ☐ 5 (extremely complex)

Complexity of solution

(timing of administration: after rehabilitation – experimental group only)

<u>Robotic rehabilitation solution</u>

1. ***What is the main problem addressed by robotic rehabilitation solutions?***
   - ☐ Self-care
   - ☐ Language
   - ☐ Balance
   - ☐ Walking
   - ☐ Manual skills
   - ☐ Other (specify): ____
2. ***Based on your point of view, how complex is this problem?***
   - ☐ 1 (not complex)
   - ☐ 2
   - ☐ 3
   - ☐ 4
   - ☐ 5 (extremely complex)
3. ***How complex are robotic rehabilitation solutions?***
   - ☐ 1 (not complex)
   - ☐ 2
   - ☐ 3
   - ☐ 4
   - ☐ 5 (extremely complex)

Computer skills

How do you rate your level of knowledge in using... (1: very low, 5: advanced)

	1<br>(very low)	2	3	4	5<br>(advanced)
Desktop computer					
Laptop					
Smartphone					
Tablet					
Other ICT devices					

Do you use any of the following technologies in your daily routine? If yes, also indicate the frequency of use:

	Never	Only once or a few times)	Once a month	Often	Daily use
Desktop computer					
Laptop					
Smartphone					
Tablet					
Other ICT devices					

Digital Skills – Information

	Yes	No
I can search for information online using a search engine.		
I know that not all online information is reliable.		
I can save or archive files and content (e.g., text, images, music, videos, web pages, ...) and retrieve them once archived		

Digital Skills - Communication

	Yes	No
I can communicate with others using a mobile phone, Voice over IP (e.g., Skype), email, or chat, using basic functionalities (e.g., voicemail, SMS, sending and receiving emails, text exchange).		
I can share files and content using simple tools.		
I know I can use digital technologies to interact with services (such as governments, banks, hospitals).		
I am aware of social networking sites and online collaboration tools.		
I am aware that certain communication rules apply when using digital tools.		

Digital Skills - Content Creation

	Yes	No
I can produce simple digital content (e.g., text, tables, images, audio files) in at least one format using digital tools.		
I can make basic modifications to content produced by others.		
I know that content can be covered by copyright.		
I can apply and modify simple functions and settings of the software and applications I use (e.g., changing default settings).		

Digital Skills – Security

	Yes	No
I can take basic measures to protect my devices (e.g., using antivirus and passwords).		
I am aware that my credentials (username and password) can be stolen.		
I know I should not disclose private information online.		
I know that excessive use of digital technology can affect my health.		

Digital Skills - Problem Solving

	Yes	No
I can find support and assistance when a technical problem occurs or when using a new device, program, or application.		
I can solve some routine problems (e.g., closing a program, restarting the computer, reinstalling/updating a program, checking the internet connection).		
I know that digital tools can help me solve problems. I am also aware that they have limitations.		
When faced with a technological or non-technological problem, I can use the digital tools I know to solve it.		

Costs Assessments Questionnaire

For each of the following items, estimate the costs directly incurred by the family in the last month related to rehabilitation.

Direct Healthcare Costs

- Purchase of prescription drugs related to the rehabilitation period: _______
- Purchase of over-the-counter drugs from the pharmacy: _______
- Tests or medical exams performed during or after consultations: _______
- Other treatments or paramedical care: _______
- Expenses for the purchase of medical devices: _______

Direct Non-Healthcare Costs

- Travel expenses for the informal caregiver to accompany you to visits or rehabilitation sessions: _______
- Average distance from your home to the visit and rehabilitation center (km): _______
- Parking expenses: _______
- Accommodation expenses for attending visits: _______
- Expenses for non-reimbursed specialist visits: _______
- Home renovation expenses to meet the patient's needs: _______
- Time spent by a formal caregiver due to limited ability to perform household activities or for home rehabilitation or to accompany the patient to the healthcare center (hours per month): _______
- Hourly wage of the formal caregiver or non-family companions: _______
- Total hours of time dedicated by the informal caregiver (family member) to assist you, outside of normal working hours: _______
- Other expenses: _______

Indirect Costs (Productivity Loss)

- Total workdays lost by all informal caregivers to assist you: _______
- Total workdays lost (if working) by you due to rehabilitation: _______
